# Chicago Society of Physicians and Surgeons

**Published:** 1875-06-01

**Authors:** E. Warren Sawyer


### Society Reports.

### CHICAGO SOCIETY OF PHYSICIANS AND SURGEONS.

REGULAR MEETING, MAY 10TH, 1875.

*Reported by E. Warren Sawyer, M.D.*

THE Society met at the Grand Pacific Hotel, the President, Dr. Bartlett, in the chair.

This being the annual meeting, the yearly report of the Secretary and Treasurer was called for and read.

The Society then proceeded to the election of officers for the ensuing year, with the following result:

President, Thomas Bevan, M.D.; Vice-President, R. C. Hamill, M.D.; Secretary and Treasurer, Ralph E. Starkweather, M. D.; Recorder, E. Warren Sawyer, M.D.; Censors, Drs. Bartlett and Hyde.

Upon retiring, the President delivered the annual address, the publication of which was called for by the Society. It will appear shortly.

REPORT OF THE SECRETARY OF THE CHICAGO SOCIETY OF PHYSICIANS AND SURGEONS, FOR THE YEAR ENDING MAY 10TH, 1875.

*Mr. President and Gentlemen:*

I have the honor to submit herewith the annual report of the Secretary of the Society, for the third year since its organization.

On the 11th of May, 1874, there were fifty-nine members in active co-operation with the Society, and ten non-residents whose names were carried on the roll. Since that date three have removed from the city and twenty-one have united with us. This leaves eighty-two names of members on the active list. One has been by vote declared an associate member. Deducting this name, with those who have ceased to reside here, from the entire number, we have seventy-eight names of physicians in and about the city who are actively interested in the work of the Society.

We have held twenty-three regular meetings during the year, and one special, making an average of two for each month, with a total attendance of 479, the average attendance upon each meeting being therefore nineteen.

The following reports have been made during the year just closed:

1. On the Absorption Bands of Blood in the Spectroscope.

2. On the Relative Mortality of Surgical Operations in the United States and other countries.

3. On the Relation of Alcoholism and Sexual Diseases to Climate.

4. On the Ætiology and Pathology of Cholera Infantum.

5. On Asiatic Cholera — being a detailed account of its ingress and extension through portions of the United States during the summer of 1873.

6. On a New Method of Introducing Barnes' Dilators.

7. On the Action of Hydrate of Chloral upon the Blood.

8. On Pneumatic Aspiration — its methods and instruments.

9. On General Therapeutics.

10. On Carcinomatous and Scirrhous Neoplasms, with microscopical illustrations.

11. On the Organic Alkaloids of the Cinchona Barks.

The following papers have been read before the Society during the year:

1. On the Microscopy of the Brains of the Insane (with illustrative drawings).

2. On the use of Podophyllin in Acute Rheumatism (pub. *Am. Jour. of Med. Sci.*).

3. On the Eucalyptus Globulus.

4. On the Relative Value of Thermometers for Medical Purposes offered to the Profession.

5. On Puerperal Convulsions, with illustrative case.

6. On the Management of the Third Stage of Labor.

7. On the Employment of Phytolacca Decandra and Guacum in Rheumatic Disorders.

8. On the Action of Salicylic Acid.

The following translations of papers by foreign authors were also presented and read:

1. On the Therapeutic Value of Ipecac in Pulmonary Sweats.

2. On the Examination of the Mucous Membrane of the Uterus (with illustrative microscopic drawings).

The following cases have been reported:

1. Case of double vagina and uterus, with successful delivery of living child.

2. Case of chronic pyelitis.

3. Case of sub-acute meningitis.

4. Case of amenorrhœa.

5. Case of dysmenorrhœa, relieved by topical application of Faradic current.

6. Cases of relapsing fevers.

7. Cases of obscure neuroses.

8. Case of retained menses from vaginal constriction, successfully relieved by surgical interference.

9. Cases from clinic for diseases of heart and lungs—South Side Dispensary.

10. Cases of sub-involution of uterus, relieved by topical applications of nitric acid—in the "Woman's Hospital," State of Illinois.

11. Case of retention of placenta during twelve days.

12. Case of suppurative pleuritis relieved by aspiration.

13. Two cases of epilepsy successfully treated.

14. Case of scirrhus of the stomach, with details of post-mortem appearances and microscopic pathology (illustrated by stained sections under the microscope).

15. Cases of uterine inertia, relieved by actæa racemosa.

16. Case of aspiration of the pericardial sac, partial relief, death, and exhibition of specimen.

17. Two cases of fracture of arm and leg (St. Luke's Hospital).

18. Case of multilocular ovarian cyst successfully treated by electricity and acipuncture.

19. Fatal case of corroding ulcer of the uterus.

20. Case of traumatic epileptiform convulsions.

21. Case of variola arrested by employment of sulphite of soda and carbolic acid.

22. Four cases of land scurvy successfully treated in Cook County Hospital.

23. Two cases of uterine fibroid tumors relieved by hypodermic injection of ergotine.

24. Case of cerebro-spinal meningitis simulating hydrophobia.

25. Case of successful removal of colloid multilocular ovarian tumor, followed by death, with chart of pulse and temperature record, and exhibition of specimen.

26. History of a singular case of catalepsy, treated in Mercy Hospital.

The following specimens have been presented during the year, in addition to those already mentioned:

1. Nine calculi removed from patient at a single operation.

2. Horn removed from the forehead of a woman fifty-five years old.

3. Portable electro-faradic machines with chloride of silver batteries.

4. Tumor of Dentine.

5. Cast of an anencephalous monster.

6. A novel inhaler for anæsthetic purposes.

During the year Art. I. of the Constitution has been amended so as to exclude candidates for membership who have not been in actual practice for two years, and who may be interested in the sale of medicines and surgical apparatus. Two sections have also been added to Article IV., providing for the election of associate and honorary members; while a new by-law has been added, restricting the privilege of each member to the nomination of but one candidate during the year.

Two important resolutions have been passed, one recommending the delegates of the Society, to both the State and National Medical Associations, to urge upon those bodies the memorializing Congress with reference to the remission of the duties on imported scientific works; the other fixing the annual meeting as the appropriate date for the appointment or election of such delegates.

In accordance with a resolution of the Society, passed Dec. 28, 1874, a committee has been appointed to wait upon the President of the Society, and, in accordance with his expressed wish, examine his specimens of the fungus from the Mississippi River ague bottoms, and report in full to the Society upon the same, as well as upon the paper prepared and read by Dr. Bartlett on the 10th of April, 1873. I am informed by the chairman of the committee that they have waited upon Dr. Bartlett and arranged upon a date for the investigation proposed.

The protracted work and subsequent report of the committee on the relations between physicians and pharmacists are fresh in your recollection, and require no word of comment from one who co-operated with them in detail. It is sufficient to note that the result obtained was all that any reasonable individual could have anticipated at the outset of the inquiry. It awakened a sentiment in the minds of many men connected with the two professions which led them to define for themselves a distinct boundary line between what should and what should not be permitted in the light of the highest ethics.

Nor is it necessary to review the part taken by this Society in the banquet extended by the medical profession of the city to the State Association at the date of its last annual convention in our midst. The large representation of the Society in the number that enjoyed the festivities of that occasion, and the able address of our President, are remembered by many who are gathered here to-day. Were the recollection wanting, the details published in the transactions of the Medical Society of the State of Illinois for 1874, would vividly recall the occasion. It is proper to add that that was the largest social gathering of the representatives of the medical profession of the city of Chicago that has ever occurred, and so great was the harmony and good feeling fostered by the amenities of the banquet, that not a few have felt it would be a desirable end accomplished if some such social reunion could occur annually.

The thanks of the Society during the year concluded are due to the editors of the two medical journals of Chicago for their kindness in publishing fully all the transactions and papers of the Society that have been submitted to them. The resolution passed Nov. 23d, 1874, requiring that thereafter only the strictly scientific and literary transactions of the association should be spread before the public, reserving for the records the executive and other acts, have improved the character of the published material.

In this connection, it is proper to state that for the accuracy and regularity with which all such matter has been prepared for publication, the Society itself, and the editors of the medical journals, no less, are greatly indebted to the faithfulness of the reporter for the year, Dr. R. E. Starkweather. When it is considered that he has been under the necessity during the entire year of preparing duplicates of each report of the transactions, one for each journal, and that these reports have frequently embodied long debates and abstracts of extended papers and verbal reports, an idea may be had of the actual labor involved in the discharge of his duties. How efficiently he has performed his task you have been in position to determine who have examined the weekly and monthly exhibits of the medical press.

To Dr. F. H. Davis, the editor and publisher of the MEDICAL EXAMINER, the Society is indebted for a bound copy of all the papers and transactions published in that journal during the year 1874. These were collected by Dr. Davis himself, from the mass of material which was at his disposal, and bound in this convenient shape for the purpose of presenting them to the Society.

In concluding this resume of the work accomplished during the past year, it will be apparent, even upon a hasty glance over the field, that the character and amount of original investigation which has been encouraged in our midst, are sufficiently gratifying. It is also sufficient to suggest the conclusion that he who has lost the opportunities afforded here for actual advance in the directions to which the reports have pointed, has failed to secure the absolute advantages provided by the Society. If some there be of this class, they have but one consolation left them, and that is the ability to offset this loss by equally solid advantages gained elsewhere. The species of moralizing which is suggested by the arrival at such anniversaries as the present, is at once natural and necessary. The question comes home to each individual upon such occasions, "Have I, in a satisfactory manner, discharged my duties—not to the Medical Society, but to myself—during the period which has elapsed?"

Now, just here comes in the operation of a principle, which is clearly admitted by all men, and yet which they are quick to lose sight of. Perhaps in consideration of the fact that to-day, after serving in the capacity of secretary for three years, I resign my office to the hand of another, I may be pardoned for a representation of it.

It is a common enough error to conclude that the benefit to be derived from an association of this kind is obtainable by attendance upon its meetings, listening to the papers read, and the discussions which result, and digesting the accumulated information so as to make it the property of the hearer. It is undeniably true that benefit is to be gained by this process, but so inferior is it to the larger improvement of another kind placed within reach of all that the end would not actually justify the expenditure of the time and trouble necessary to secure it.

The most valuable of the advantages afforded by a medical society are certainly *not* those resulting from the information to be acquired at its meetings. For the periodicals and permanent literature which provide the student with the fullest details upon every question, are accessible to each in the office and study. What extempore speaker among us can furnish such instructive pabulum as Murchison, Broadbent, Biesiodecki, or Nelaton? What essayist of our number can compare in brilliancy and depth with Hutchinson, Bennett, Bernutz, or Virchow? Shall we then abandon our efforts, and cease to unite ourselves in the bands of medical organizations?

The answer given by the thoughtful man will be a decided negative, and his reason will be this: The greatest benefit derivable from these associations accrues to the speaker, the writer, the exhibitor, and *not* to the hearer. It is he who, having observed and amassed facts, comes to others with his treasure, and toils in the effort; it is he, I say, who obtains the great benefit of the deed, not he merely who receives his share of the treasure. It is not the *receiving* which makes us richer; it is the giving. He who brings me a fact or an idea is richer than I. It is the gladiator in the arena whose muscles are hardened, whose courage is heightened, whose brain becomes cool and clear, not the listless or interested spectator on the benches.

The American poet, Lowell, has illustrated the truth of this universal law in one of the most finished of his productions. In the "Legend of Sir Launfal," the knight goes forth in quest of the Holy Grail, but returns at the conclusion of a vain and weary pilgrimage, utterly disheartened. It is only when he is quite near his home, and divides his scanty crust with a miserable beggar that he is rewarded by discovering the sacred object of his search.

The knight of the helmet, spear, and shield, has long been succeeded by the man with the microscope, the thermometer and the scalpel. But the latter, no less than the former, will fail to attain the precious end of his researches, if he does not divide by the way his crust of truth with his fellow.

That member of this Society who faithfully discharges his duty to his associates during the ensuing year, will assuredly be the wiser and the stronger for the effort.

JAMES NEVINS HYDE.

---

